# *In vivo* Assessment of Localised Corneal Biomechanical Deterioration With Keratoconus Progression

**DOI:** 10.3389/fbioe.2022.812507

**Published:** 2022-06-08

**Authors:** Bernardo T. Lopes, Prema Padmanabhan, Ashkan Eliasy, Haixia Zhang, Ahmed Abass, Ahmed Elsheikh

**Affiliations:** ^1^ School of Engineering, University of Liverpool, Liverpool, United Kingdom; ^2^ Department of Ophthalmology, Federal University of São Paulo, São Paulo, Brazil; ^3^ Department of Cornea and Refractive Surgery, Sankara Nethralaya, Chennai, India; ^4^ School of Biomedical Engineering, Capital Medical University, Beijing, China; ^5^ Department of Production Engineering and Mechanical Design, Faculty of Engineering, Port Said University, Port Fuad, Egypt; ^6^ Beijing Advanced Innovation Center for Biomedical Engineering, Beihang University, Beijing, China; ^7^ National Institute for Health Research (NIHR) Biomedical Research Centre for Ophthalmology, Moorfields Eye Hospital NHS Foundation Trust and UCL Institute of Ophthalmology, London, United Kingdom

**Keywords:** biomechanics, keratoconus, progression, SSI, corvis, stress strain index

## Abstract

**Purpose:** To evaluate the regional corneal biomechanical deterioration with keratoconus (KC) progression as measured by the Stress-Strain Index (SSI) maps.

**Methods:** The preoperative examinations of 29 progressive KC cases that were submitted to corneal cross-linking (CXL) were evaluated. The examinations included the tomography and the SSI measured by the Pentacam HR and the Corvis ST (Oculus, Wetzlar, Germany), respectively. The results were recorded twice, the latter of which was at the last visit before the CXL procedure. The patient-specific SSI maps were built, using data at each examination, based on finite element modelling and employing inverse analysis to represent the regional variation of biomechanical stiffness across the cornea.

**Results:** All cases presented significant shape progression (above the 95% CI of repeatability) in anterior and posterior curvatures and minimum thickness. The overall corneal stiffness as measured by the SSI within the central 8 mm-diameter area underwent slight but significant reductions from the first to the last examination (−0.02 ± 0.02, range: −0.09 to 0, *p* < 0.001). In all 29 cases, the reduction in stiffness was localised and concentred in the area inside the keratoconus cone. The SSI values inside the cone were significantly lower in the last examination (by 0.15 ± 0.09, range: −0.42 to −0.01, *p* < 0.001), while the SSI outside the cone presented minimal, non-significant variations (0 ± 0.01, range: −0.04 to 0.01, *p* = 0.999).

**Conclusion:** It has been observed through the SSI maps that the regional deterioration in stiffness was concerted inside the area of pathology, while only mild non-significant alterations were observed outside the area of pathology.

## Introduction

**FIGURE 1:**
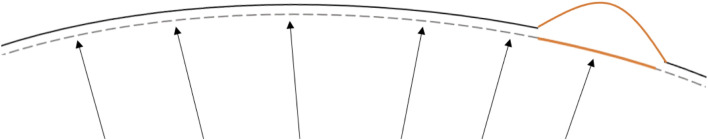
Schematic representation of localised corneal softening (orange) that under the same intraocular pressure load would deform in greater extent than the surrounding stiffer areas leading to the characteristic corneal ectatic bulging. The dashed line represents the corneal stress-free state. The arrows, a schematic representation of the intraocular ocular pressure and the continuous line the deformed state induced by the intraocular pressure.

Keratoconus (KC) starts with a localised biomechanical weakening of corneal tissue that progresses to shape deformation and vision deterioration (Mas Tur et al., 2017). The bulging process of corneal tissue occurs when the localised diseased area (with a lower elastic modulus) deforms more than the surrounding healthy tissue (with a higher elastic modulus) when loaded with the intraocular pressure (Figure 1) (Roberts and Dupps, 2014). Recent developments in ophthalmic devices allowed the measurement of corneal biomechanics *in vivo* and potentially a better evaluation of disease progression (Esporcatte et al., 2020).

In the past, due to the absence of direct measures, corneal biomechanical deterioration was assessed predominantly through corneal shape alterations, which develop at a late stage of disease pathophysiology (Brown et al., 2014). A common attempt made by the clinicians to infer the corneal biomechanical state and evaluate the ectasia risk in clinics was by means of surrogates such as age (Ambrósio et al., 2014). Ferdi et al., in a metanalysis on KC natural progression, observed that a steeper baseline corneal curvature and younger age were the two main factors associated with disease progression (Ferdi et al., 2019). The authors suggested that a closer follow-up and a low threshold for CXL indication should be adopted in patients younger than 17 years old or with KMax higher than 55 D. As both criteria are thought to be related to softer corneal tissue, Ferdi’s recommendations seem to be in line with KC pathophysiology (Mas Tur et al., 2017).

Some advances to measure the corneal biomechanical properties *in vivo* and improve corneal ectatic diseases management began in the early XXI century. The Ocular Response Analyzer (ORA, Reichert, New York, United States), was the first device to provide corneal biomechanics-related measurements. Initially, it offered two parameters; the Corneal Hysteresis (CH) and the Corneal Resistance Factor (CRF), which reportedly were correlated with the tissue’s viscoelasticity and stiffness, respectively (Luce, 2005; Pepose et al., 2007). It was followed by the development of the Brillouin microscopy that assesses the corneal longitudinal modulus *in vivo* and the Corvis ST (Oculus, Wetzlar, Germany), which provides a number of Dynamic Corneal Response (DCR) parameters derived from the deformation of the central corneal horizontal profile under an air-puff pressure (Lopes B. T. et al., 2021). Both the ORA and the Corvis ST did not initially produce indices that could be directly related to standard biomechanical properties, even though their biomechanical metrics had clear links with corneal deformation behaviour, KC progression signs, and ectasia risk assessment (Fontes et al., 2010; Luz et al., 2016; Vinciguerra et al., 2016; Ambrósio et al., 2017). The Corvis ST parameters have also been shown to shift towards stiffening after the CXL procedure (Vinciguerra et al., 2017). Another *in vivo* technique developed to measure the corneal biomechanics *in vivo*, the Brillouin microscopy, provides an estimate of the corneal longitudinal modulus (given that the material’s refractive index and density are known) (Prevedel et al., 2019) in spatially resolved maps (Shao et al., 2019), but lacks consideration of the tissue’s hyperelasticity and viscoelasticity.

The more recent development of the Corvis ST, and the stress-strain index (SSI), overcomes the initial limitation of the absence of a direct link of the exam variables with standard mechanical parameters (Eliasy et al., 2019). The index provides an estimation of the overall corneal stress-strain behaviour that considers the corneal non-linear behaviour, therefore allowing the estimation of the tangent modulus (Et) at any load or stress (Roberts and Dupps, 2014).

The assumption of homogeneity embedded in the SSI development, however, can mask the localised biomechanical deterioration that takes place in KC. To solve this problem, Zhang et al. proposed a method to map SSI across the cornea based on known trends in collagen fibril density distribution and how these trends change in keratoconic corneas (Zhang et al., 2021). This method is of particular importance in evaluating disease progression. The development of corneal crosslinking (CXL) to treat KC progression has generated a need to detect early signs of the disease so that the procedure be offered in good time to preserve the patients’ clear vision (Kobashi and Rong, 2017). Also, as CXL is not an innocuous procedure, demonstration of corneal biomechanical stability and lack of disease progression would prevent unnecessary treatment, avoiding risks of iatrogenic complications (Evangelista and Hatch, 2018).

This study aimed to apply the SSI mapping method for the first time in progressive KC cases and understand how the stiffness deterioration takes place in this scenario.

## Methods

### Clinical Examinations

A fully anonymised database of keratoconic patients that have undergone crosslinking surgery at the Sankara Nethralaya Eye Hospital (Chennai, India) was reviewed retrospectively. For this anonymised record review study, approval was ruled unnecessary by the local institutional review board. Nonetheless, the ethical standards set in the Declaration of Helsinki and its subsequent revisions were observed. Every patient provided signed informed consent for the use of their de-identified clinical data in research.

The inclusion criteria were the presence of complete records at the preoperative period including complimentary exams of corneal tomography with the Pentacam HR and biomechanical assessment with the Corvis ST (Both, Oculus, Wetzlar, Germany), and the presence of clear KC progression. Each examination was manually analysed frame-by-frame by an independent masked examiner (AA) to ensure the quality of each acquisition, including the absence of blinking errors or alignment errors while ensuring good edge detection over the whole deformation response or rotating Scheimpflug images. The exclusion criteria were defined as the presence of previous ocular surgeries or corneal diseases other than keratoconus.

The KC progression was considered according to the statements of the Global Consensus in Keratoconus and Ectatic Diseases, in which the steepening in the anterior or posterior corneal surfaces or corneal thinning should be above the systematic noise of the testing system (Gomes et al., 2015). In light of this recommendation, Pentacam’s ABCD staging classification system was used. The 95% confidence interval (CI) of the repeatability in KC cases was used to assess the changes in the anterior radius of curvature (ARC), the posterior radius of curvature (PRC) and minimum corneal thickness. For cases that presented changes above the 95% CI, the tomographic and biomechanical exams of two-time points, the first and the last visits before the CXL procedure were reviewed. The mean time between the two examinations was 17.1 ± 17.1 months (1.4–58.4). The biomechanically corrected intraocular pressure (bIOP) and the corneal deformation behaviour were assessed from the Corvis ST.

### SSI Maps

**FIGURE 2:**
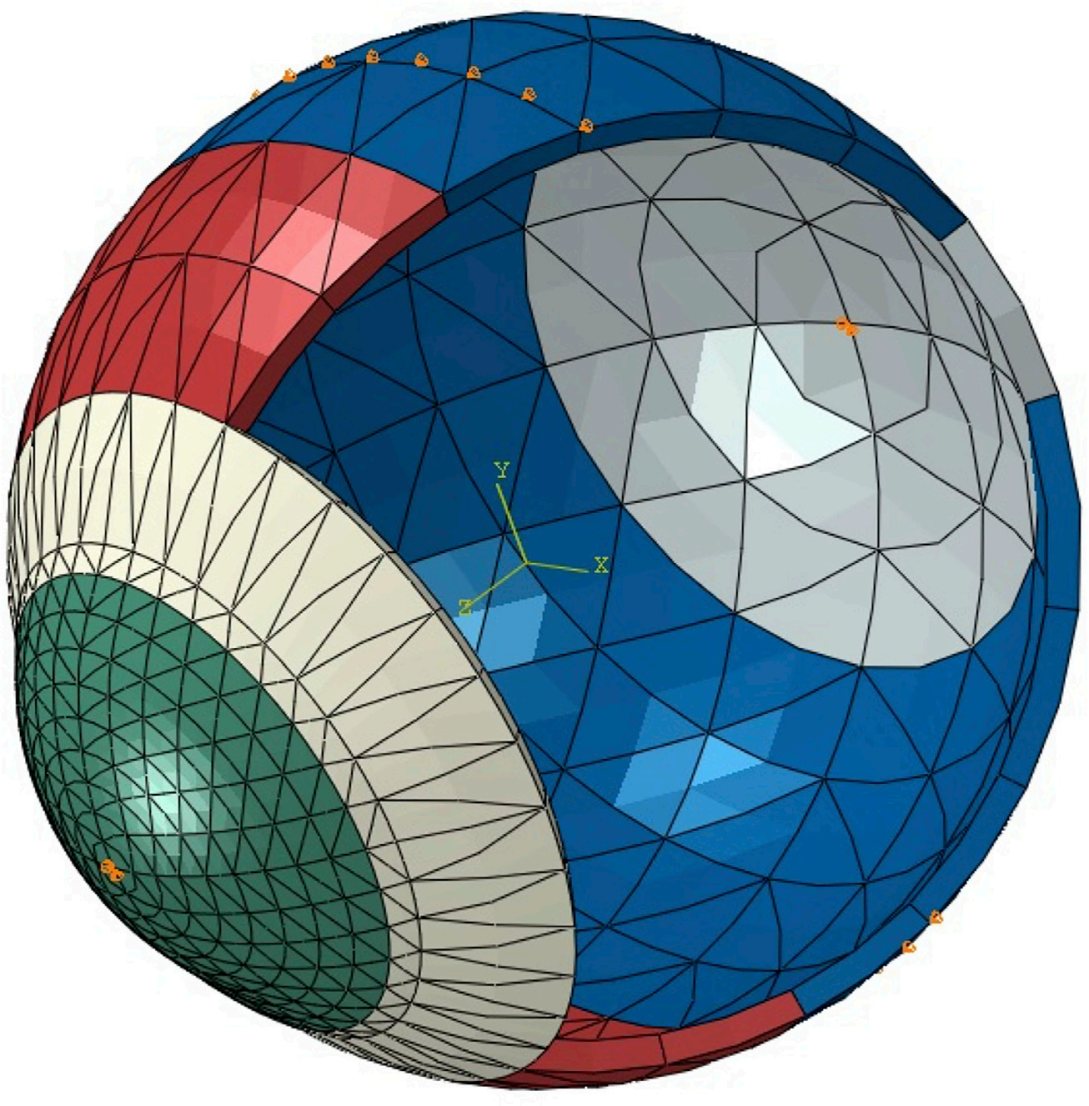
A typical whole-eye finite element model showing the boundary conditions applied at the equator and corneal apex (orange pins).

The development of SSI maps for individual corneas followed the method proposed by Zhang et al. to convert the single SSI value obtained from the Corvis exam into a 2D map (Zhang et al., 2021). In brief, the process begins by estimating three KC cone features: apex location, cone height and KC area of pathology, based on a method proposed by Eliasy et al. that was later clinically validated (Eliasy et al., 2020; Lopes B. et al., 2021). The fibril density reduction inside the cone was then estimated based on these KC cone features using the method proposed by Zhou et al. (Zhou et al., 2021). Using the tomography data of the corneas under study, eye-specific 3D finite element (FE) models were built using fifteen-noded continuum solid elements, described in detail, including an integrated mesh convergence study, elsewhere (Eliasy, 2021). In brief, as observed in Figure 2, the elements were organized in a single layer and in rings, where the number of rings controls the mesh density. To determine the optimal number of corneal rings that would maintain consistent outputs by the model, while using the smallest possible number of elements to optimise analysis cost, models with 5–45 corneal rings, in steps of 5, were generated. All models initially had 35 scleral rings and were subjected to inflation and air pressure. From 5 to 10 rings there was an 11% increase in the apical deformation from 722 to 800 μm, followed by much smaller changes, below 10 μm, with denser corneal meshes. Therefore the 10-ring model was chosen for the cornea. Then, in order to determine the optimal number of scleral elements, new models with the number of corneal rings fixed at 10 and the number of scleral rings varied between 10 and 50, in steps of 10, were generated and tested under inflation and air pressure. The variation in corneal apical deformation in all six models was limited to 1 μm, allowing selection of 10 sclera element rings in the final model. With these decisions, the optimal mesh included 800 elements organised in 10 corneal and 10 scleral rings, Figure 2. Further, rigid body motion of the models was prevented by restricting motion at the scleral equatorial nodes in the axial direction, and the corneal apex in both the temporal–nasal and superior-inferior directions. The aqueous and vitreous humour were simulated by a fluid cavity filled with an incompressible fluid with a density of 1,000 kg/m^3^. Through utilizing the corneal internal fluid pressure, intraocular pressure (IOP) was applied. The Corvis air pressure was then applied to the cornea of the pressurised eye causing the corneal tissue to deform in response. Two different material models were adopted in two otherwise-identical eye models to conduct an inverse analysis process In Model 1, a third-order, hyper-elastic Ogden homogeneous material model, whose stiffness at each integration point originated from the corresponding SSI value—as described in an earlier study (Eliasy, 2021). In Model 2, a constitutive material model that used the collagen fibril distribution to control the regional and angular variation of stiffness across the cornea, as described by Zhou et al. was adopted (Zhou et al., 2019). The latter constitutive model was coded using a custom-built subroutine, integrated with the analysis process run on Abaqus FE software (version 6.14, Dassault Systemes Simulia Inc., United States). In Model 2, different integration points adopted stiffness levels, the ratios between which corresponded to the ratios between fibril content at these points. An inverse analysis was then carried out in order to minimize the differences in corneal apical displacements of the two models under Corvis air-puff pressure. The analysis adopted the following objective function in which the root mean squared error (RMSE) indicated the mismatch between the apical displacements ( δ ) of Models 1 and 2:

$$Root\;mean\;square\;error\;\left(RMSE\right)=\;\sqrt{\frac{\sum_{i=1}^{n}{\left(\delta_{i\;Model\;1}-\;\delta_{i\;Model\;2}\right)}^{2}}{n}}$$

where *n* is the number of data points.

When the RMSE reached its smallest possible value, the corresponding distribution of SSI across the numerical model was taken as the SSI map of the cornea.

### Statistical Analysis

Statistical analyses were accomplished using the R Core Team (2016, R Foundation for Statistical Computing, Vienna, Austria). The mean SSI values inside the cone, outside the cone and its overall value in the central 8 mm-diameter area were calculated and compared between the first and last exams. Due to the absence of normal distribution in the evaluated variables, the non-parametric Wilcoxon signed-rank test was used to compare the paired data. Boxplots were used to assess the distribution of each variable between the two exams. Statistical significance was considered if *p* < 0.05.

## Results

The 29 eyes included in the study presented keratoconus in different stages with maximum anterior curvature (KMax) of 54.37 ± 4.55 D (44.5–64.4) and minimum thickness of 468.55 ± 27.74 µm (414–520). The mean age at the last exam before CXL was 20.1 ± 7.0 years (9–40). The right to left eye ratio was 1:1.2. The mean time between the two examinations was 17.1 ± 17.1 months (1.4–58.4). Significant worsening in corneal shape, represented by anterior steepening (ARC, −0.42 ± 0.29 mm, *p* < 0.001), posterior steepening (PRC, −0.40 ± 0.25 mm, *p* < 0.001) and reduction of minimum corneal thickness (−26.17 ± 17.94 µm, *p* < 0.001), was observed between the two exams. Despite these progression signs, little not-significant change was observed in the KC area of pathology (−0.2 ± 0.9 mm^2^, *p* = 0.156).

**FIGURE 3:**
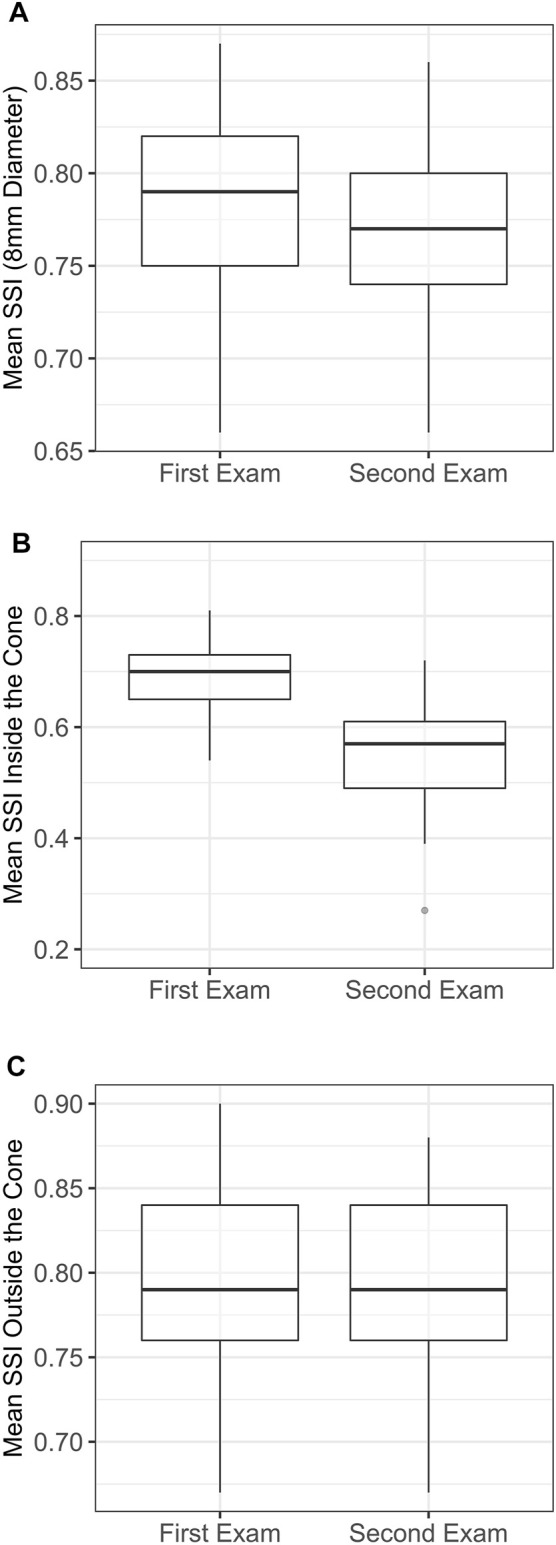
Localised distribution of the SSI in each examination. **(A)** Mean SSI over 8 mm diameter. **(B)** Mean SSI inside the cone area. **(C)** Mean SSI outside the cone area. SSI, stress-strain index.

**FIGURE 4:**
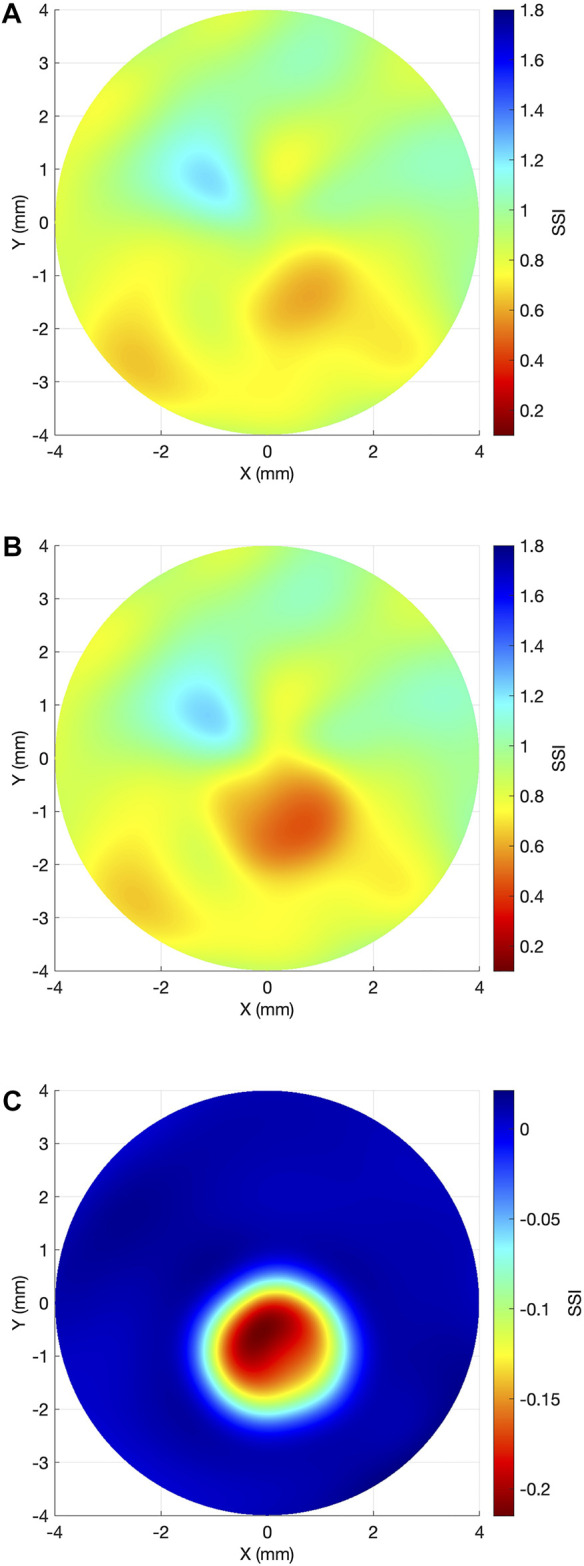
Representative case example of SSI map change in keratoconus progression. **(A)** Baseline SSI map. **(B)** SSI map after 25 months. **(C)** Differential SSI map.

The overall SSI value in the central 8 mm-diameter area presented a slight but significant reduction from the first to the last exam (−0.02 ± 0.02, range: −0.09 to 0, *p* < 0.001). Meanwhile, the SSI values inside the KC area of pathology underwent significant reductions in the last exam (−0.15 ± 0.09, range: −0.42 to −0.01, *p* < 0.001), while the SSI outside the KC area of pathology presented minimal, non-significant differences (0 ± 0.01, range: −0.04 to 0.01, *p* = 0.999). Minimum non-significant alteration between the two exams was also observed in the bIOP (−0.08 ± 1.21 mmHg, range: −2.0 to 2.3, *p* = 0.584). Figure 3 illustrates these results. In Figure 4, a representative example of SSI maps obtained for a 21-year-old male patient with moderate KC that progressed over 25 months with an increase in KMax from 54.7 D to 56.2 D, increase in posterior elevation at the thinnest point from 49 to 70 µm and corneal thinning from 504 to 491 µm.

## Discussion

In the present study, the regional variation in corneal stiffness that occurs with KC progression was assessed for the first time *in vivo* using the SSI Maps. In a disease like KC, which softens a portion of the cornea, leaving the remaining tissue relatively unaltered, having the ability to map local areas of softening in the corneal tissue presents a great advantage to its clinical management. It can allow the indication of treatment with CXL to halt disease progression before serious visual deterioration is developed with a potential impact on the patient’s quality of life (Yildiz et al., 2021).

The results showed that even though, there was a small but significant reduction in the overall SSI between the two exams (−0.02 ± 0.02, *p* < 0.001), this reduction was mainly concentrated inside the diseased area (−0.15 ± 0.09, *p* < 0.001), while outside it, there was no significant change between the exams (0 ± 0.01, *p* = 0.999). These results are in line with *ex vivo* experimental results using electron microscopy and x-ray scattering in which the collagen fibrils’ organisation was significantly altered inside the keratoconic KC area of pathology but remained unaffected outside it (Radner et al., 1998; Meek et al., 2005). Using x-ray scattering, Zhou et al. observed that the magnitude of collagen fibril density reduction inside the cone area was up to 35% in advanced KC cases (Zhou et al., 2021).

Also in the same line, Scarcelli et al. in an *ex vivo* study using Brillouin Microscopy in healthy and KC corneas observed that the Brillouin frequency shift within the cone region (7.99 ± 0.10 GHz) was significantly lower than those observed in corresponding areas of healthy corneas (8.17 ± 0.06 GHz, *p* < 0.001) (Scarcelli et al., 2014). Within the KC specimens, the Brillouin shift was significantly higher in areas outside the cone (8.19 ± 0.04 GHz, *p* < 0.001), and these values were not significantly different from those observed in healthy corneas (*p* > 0.05) (Scarcelli et al., 2014).

The BM was also used *in vivo* healthy and KC subjects with similar results: significant reduction in the Brillouin frequency shift within the cone region in KC cases when compared, in the same case, to areas outside the cone or to the equivalent areas in healthy subjects (Scarcelli et al., 2015). A progressive increase in the Brillouin shift was observed from the cone region towards the corneal periphery and the rate of regional variation increased with disease severity (Shao et al., 2019). However, to the best of our knowledge, no longitudinal study has been performed to evaluate the effect of the intrasubject effect of KC progression in the Brillouin Microscopy 3D maps.

A better understanding of the biomechanical changes with KC progression could allow the improvement of CXL treatment. This could be done by a timely indication of the procedure or by customising the procedure to the patient’s biomechanical needs. A few attempts have been made to customise the CXL treatment. Kling and Hafezi developed an algorithm to predict the biomechanical stiffening effect of the procedure (Kling and Hafezi, 2017). It has been successfully used to adjust the treatment fluence in high-risk patients with corneal thickness below 400 μm, halting the disease progression in 90% of the cases after 1 year (Hafezi et al., 2021). Other attempts to customise the treatment adjusted the irradiance pattern according to each case individual topography with reported success in stabilizing the disease that was comparable to the standard procedure but with improved flattening of the maximum corneal curvature (Seiler et al., 2016; Cassagne et al., 2017). However, the previous attempts did not focus on the underlying biomechanical degradation that succeeds with the disease progression and is ultimately responsible for the topographical changes.

A few limitations to this study are discussed here. The study relied on numerical modelling, which while being validated and optimised, may still include approximations in geometry and material behaviour. For example, the model considers the tissue’s behaviour to be consistent through the thickness, which is not compatible with current evidence that the anterior part of the stroma is stiffer than the posterior part (Dias and Ziebarth, 2013). This approximation, which was necessary due to the lack of complete information on the through-thickness variation in behaviour, could have influenced the model’s outputs. In the numerical modelling used to develop the SSI maps, it was assumed that the areas outside the cone in keratoconic corneas shared the same microstructure as corresponding areas in healthy corneas (Zhang et al., 2021). This assumption was necessary due to the lack of methods to measure corneal microstructure *in vivo* and was based on evidence from two earlier studies (Boote et al., 2003; Zhou et al., 2019). The first study reported only small inter-individual variations in collagen fibril content in healthy corneas of up to 2.9% (Zhou et al., 2019), and the second reported little variation among individuals in fibril diameter (Dias and Ziebarth, 2013). Another limitation is that the validation of the mapping method relied on comparing the numerically-predicted, SSI-based deformation of the cornea along only the cornea’s principal temporal-nasal meridian to that measured clinically by the Corvis ST (Zhang et al., 2021). Further, the method to relate the fibril density reduction inside the cone with the cone morphology was based on information obtained from only 7 KC corneas (Zhou et al., 2021). On the clinical side, it is important to note that the relatively small sample used in the study and the lack of cases in which there was a small progression extent represented other limitations, which will be addressed in prospective studies.

In summary, this study presents the outcome of a new *in vivo* method using FE simulation to map corneal stiffness in progressive keratoconic patients. Corroborating what has been seen in previous experimental and clinical studies, the biomechanical deterioration of these progressive patients was localised in the KC area of pathology, what suggests that the SSI maps are a reliable method to map the corneal stiffness deterioration in progressive KC. The ability to map the disease progression *in vivo* may therefore have important implications in the indication and customisation of treatments, such as corneal cross-linking.

## Data Availability

The data analysed in this study is subject to the following licenses/restrictions: There is a material transfer agreement in place. Requests to access these datasets should be directed to PP, drpp@snmail.org.
